# Strategies to Rescue Mesenchymal Stem Cells (MSCs) and Dental Pulp Stem Cells (DPSCs) from NK Cell Mediated Cytotoxicity

**DOI:** 10.1371/journal.pone.0009874

**Published:** 2010-03-31

**Authors:** Anahid Jewett, Aida Arasteh, Han-Ching Tseng, Armin Behel, Hobie Arasteh, Wendy Yang, Nicholas A. Cacalano, Avina Paranjpe

**Affiliations:** 1 Division of Oral Biology and Medicine, The Jane and Jerry Weintraub Center for Reconstructive Biotechnology, UCLA School of Dentistry and Medicine, University of California Los Angeles, Los Angeles, California, United States of America; 2 Department of Radiation Oncology, The Jonsson Comprehensive Cancer Center (JCCC), UCLA School of Dentistry and Medicine, University of California Los Angeles, Los Angeles, California, United States of America; Centre de Recherche Public de la Santé (CRP-Santé), Luxembourg

## Abstract

**Background:**

The aim of this paper is to study the function of allogeneic and autologous NK cells against Dental Pulp Stem Cells (DPSCs) and Mesenchymal Stem Cells (MSCs) and to determine the function of NK cells in a three way interaction with monocytes and stem cells.

**Methodology/Principal Findings:**

We demonstrate here that freshly isolated untreated or IL-2 treated NK cells are potent inducers of cell death in DPSCs and MSCs, and that anti-CD16 antibody which induces functional split anergy and apoptosis in NK cells inhibits NK cell mediated lysis of DPSCs and MSCs. Monocytes co-cultured with either DPSCs or MSCs decrease lysis of stem cells by untreated or IL-2 treated NK cells. Monocytes also prevent NK cell apoptosis thereby raising the overall survival and function of NK cells, DPSCs or MSCs. Both total population of monocytes and those depleted of CD16^+^ subsets were able to prevent NK cell mediated lysis of MSCs and DPSCs, and to trigger an increased secretion of IFN-γ by IL-2 treated NK cells. Protection of stem cells from NK cell mediated lysis was also seen when monocytes were sorted out from stem cells before they were added to NK cells. However, this effect was not specific to monocytes since the addition of T and B cells to stem cells also protected stem cells from NK cell mediated lysis. NK cells were found to lyse monocytes, as well as T and B cells.

**Conclusion/Significance:**

By increasing the release of IFN-γ and decreasing the cytotoxic function of NK cells monocytes are able to shield stem cells from killing by the NK cells, resulting in an increased protection and differentiation of stem cells. More importantly studies reported in this paper indicate that anti-CD16 antibody can be used to prevent NK cell induced rejection of stem cells.

## Introduction

Previous reports demonstrated significant immunomodulatory effect of Mesenchymal Stem Cells (MSCs) on different immune effector subsets such as cytotoxic T cells and Natural Killer (NK) cells [1], [2], [3], [4], [5]. Several reports have also indicated the immunosuppressive nature of MSCs on NK cells [4], [6], [7]. Moreover, activated NK cells were shown to lyse MSCs significantly. In particular, both allogeneic and autologous MSCs were shown to be targets of NK cell mediated lysis [8], [9]. Therefore, on one hand the MSCs were shown to be immunosuppressive and on the other they were found to be sensitive to lysis by the NK cells. Thus, these diverse results prompted us to evaluate the function of NK cells against two different types of stem cells, namely DPSCs and MSCs. Although some information is known about the function of NK cells against MSCs, no studies have been performed thus far to determine the effect of NK cells on DPSCs. DPSCs were shown to form colonies in vitro, and they were capable of osteogenic, chondrogenic and adipogenic differentiation [10]. In order to find ways to protect stem cells from lysis by the NK cells we need to first determine the magnitude and the mechanisms by which NK cells lyse stem cells and second to implement strategies based on those findings to protect stem cells from NK cell lysis.

Many diverse mechanisms were hypothesized to mediate the immunosuppressive effect of MSCs. TGFβ and Hepatocyte Growth Factor (HGF) were both shown to be the mediator of T cell suppression by MSCs [11]. Leukemia Inhibitory Factor induced in co-cultures of MSCs and lymphocytes was shown to mediate suppression of T cell proliferation [12]. IFN-γ induced by T cells increased B7H1 inhibitory co-stimulatory receptors on MSCs and resulted in the suppression of T cells [13]. Immunosuppressive function of MSCs is elicited by the combination of IFN-γ, TNF-α, IL-1α and IL-1β cytokines and caused elevation in the levels of chemokines and inducible nitric oxide (iNOS) [14]. Finally, indoleamine 2,3-dioxygenase (IDO) and Prostaglandin E2 (PGE2) were also shown to be the key mediators of MSC inhibition of NK cells [6]. Overall, these studies indicated the inhibitory role of key factors induced during the interaction of MSCs with the immune effectors.

We have previously coined the term of “split anergy” for the responses observed by NK_DC_ (NK cells dissociated from tumor conjugates) and NK_C_ (NK cells not dissociated from the tumor conjugate). Whereas NK_DC_ responded to IL-2 activation for cytotoxicity, they were unresponsive to IL-2 mediated induction of proliferation or secretion of cytokines. In contrast, NK_C_ showed an inverse response namely, they did not respond to IL-2 activation for cytotoxicity, but proliferated and secreted cytokines [15], [16]. Treatment of NK cells with IL-2 and anti-CD16mAb also induced split anergy by significantly decreasing the NK cell cytotoxicity while increasing the cytokine secretion capabilities of NK cells [17], [18], [19], [20]. Furthermore, IL-2 rescued anti-CD16 mAb mediated apoptosis induced in a subset of NK cells [19]. Loss of cytotoxicity in NK cells was exacerbated when NK cells were either treated with F(ab)'_2_ fragment of anti-CD16 mAb or treated with a combination of MHC-Class I and anti-CD16 mAb while the same treatments resulted in an increased secretion of cytokines [17], [20]. These results suggested that increase in signaling load on NK cells is likely to result in a decrease in cytotoxicity while increasing secretion of cytokines by the NK cells. Therefore, three distinct functional outcomes could be observed in NK cells which have either interacted with sensitive tumor-target cells or treated with anti-CD16 mAb in the presence of IL-2 treatment, namely; 1-Loss of cytotoxicity, 2-gain in the ability to secrete cytokines and 3- death in a subset of NK cells. Whether these functions are carried out by the same NK cell subsets or there are distinct subsets of NK cells with distinct functional capabilities require further studies. However, similar to T cells, NK cells can also be functionally anergized or induced to undergo cell death during increased signaling load.

Increased activity of NFκB in tumor cells was found to be inhibitory for the function of NK cells since blocking NFκB in tumor cells made these cells susceptible to lysis by the NK cells [21], [22]. Therefore, to determine which subpopulations of immune effectors were able to protect DPSCs/MSCs against NK cell mediated lysis, we undertook studies to first identify the subset of immune effectors, which was able to increase NFκB in stem cells substantially, and to determine their role in the overall increase in the survival and function of stem cells.

It is quite well established in tumor immunotherapy that certain effectors of the myeloid arm of the immune system induce resistance in tumor cells and cause suppression of NK cell mediated cytotoxicity [23]. Monocytes, an important subset of the myeloid arm of the immune effectors are known to be the major inducers of resistance in tumor cells and are shown to significantly aid in the progression of cancer in tumor-bearing hosts [23], [24], [25]. However, the mechanisms by which monocytes contribute to the progression of cancer have not been fully elucidated yet. Therefore, studies reported in this paper may also have the potential to shed light on this very important question. Our recent findings indicated that monocytes protect oral tumors via NFκB dependent and independent pathways (manuscript submitted). In addition, the role of monocytes in suppression of NK cell mediated cytotoxicity against MSCs/DPSCs has not been explored yet. Furthermore, whether a unique subset or all monocytes mediate suppression of NK cell mediated cytotoxicity against MSCs needs to be explored. Studies reported in this paper are significant in several ways. First, we demonstrate that both allogeneic and autologous NK cells are capable of lysing stem cells significantly and that lysis can be blocked by the treatment of NK cells with anti-CD16 antibody. Second, either the total populations of monocytes or those depleted of CD16^+^ subsets are capable of increasing survival of DPSCs/MSCs against NK cells in three way interactions, raising the overall secretion of key cytokines responsible for the resistance and differentiation of stem cells and protection against NK cell mediated cytotoxicity. Therefore, the effect of monocytes on NK cells are reminiscent of the effect of anti-CD16 mAb or target mediated induction of split anergy in NK cells [16], [19]. Thus, the protection of stem cells against NK cell lysis is partly due to the direct induction of resistance of stem cells by monocytes and indirectly by serving as a shield to protect lysis of stem cells by the NK cells since they too are greatly susceptible to NK cell mediated lysis in a three way interaction.

Finally, by delineating the optimal cell-cell interaction required for the protection of stem cells from NK cell mediated cytotoxicity, we can design strategies to successfully implant either allogeneic or autologous stem cells in different tissue engineering applications.

Overall, our studies demonstrate that monocytes are important catalysts of survival for different cellular subsets, and protect stem cells from NK cell mediated lysis, thereby elevating the collective functions of the cells.

## Materials and Methods

### Cell Lines, Reagents, and Antibodies

HEp2 cells were obtained from ATCC and were maintained in Dulbecco's Modified Eagle Medium (DMEM) supplemented with 10% FBS. MSCs were purchased from Clonetics and cultured with the basal medium provided by the manufacturer. DPSCs were isolated as described previously [26] and they were cultured in complete DMEM supplemented with 10% FBS. Recombinant IL-2 was obtained from the NIH repository. The NK, CD16^+^ and CD16^−^ monocyte purification kits were obtained from Stem Cell technologies (Stem Cell, Vancouver, Canada). The ELISA kits for IFN-γ, VEGF and IL-6 were purchased from R&D Systems (Minneapolis, MN). The TNF-α ELISA was developed in our laboratory and reported previously [19]. The multiplex cytokine array kit was purchased from R&D Systems. The Fluorescein Isothiocyanate (FITC) conjugated Annexin V/Propidium Iodide (PI) kit was purchased from Coulter Immunotech (Miami, FL).

### Purification of NK cells, Total monocytes and CD16^−^ Monocytes

PBMCs from healthy donors were isolated as described before [16]. Briefly, peripheral blood lymphocytes were obtained after Ficoll-hypaque centrifugation and adherence to the plate for 1 hour. Purified NK cells were negatively selected by using an NK cell isolation kit. The purity of NK cell population was found to be greater than 90% based on flow cytometric analysis of anti-CD16 antibody stained cells. The levels of CD3^+^ T cell staining in purified population of NK cells remained low at 2.4%±1%, similar to that obtained by the non-specific staining using isotype control antibody throughout the experimental procedures. The adherent subpopulation of PBMCs was detached from the tissue culture plates and the total population of monocytes and those depleted of CD16^+^ subsets (CD16^−^) were purified using isolation kits obtained from Stem Cell Technologies (Vancouver, Canada). Greater than 95% purity was achieved for each subset based on flow cytometric analysis of CD14 and CD16 antibody stained monocytes. Written informed consents approved by UCLA Institutional Review Board (IRB) were obtained from the blood donors and all the procedures were approved by the UCLA-IRB.

### ELISA and Multiplex Cytokine Array kit

Single ELISAs were performed as described previously [13]. Fluorokine MAP cytokine multiplex kits were purchased from R&D Systems (Minneapolis, MN) and the procedures were conducted as suggested by the manufacturer. To analyze and obtain the cytokine concentration, a standard curve was generated by either two or three fold dilution of recombinant cytokines provided by the manufacturer. Analysis was performed using the Star Station software.

^51^Cr release cytotoxicity assay: The ^51^Cr release assay was performed as described previously [22]. Briefly, MSCs or DPSCs were co-cultured with irradiated (10 Gy) subsets of monocytes for 24–48 hours before they were labeled with ^51^Cr for 1 hour, after which they were washed and added to NK samples. In several experiments monocytes were sorted out from the stem cell co-cultures before they were labeled with ^51^Cr and added to the NK cells. 100% removal of monocytes from the stem cells was achieved when the sorted samples were checked either by microscopy or by flow cytometric analysis of stained cells. After a 4 hour incubation period, the supernatants were harvested and counted for released radioactivity. The percentage of cytotoxicity was calculated as follows:
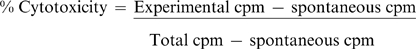



LU 30/10^6^cells were calculated using the inverse of the number of effector cells needed to lyse 30% of tumor target cells X100.

### Surface and DNA Staining and apoptosis assay

Staining was performed by labeling the cells with antibodies or propidium iodide and Annexin V as described previously [19] [16], [27].

### Luciferase reporter assay

Transfections were performed using NFκB Luciferase reporter vector [28] and Lipofectamine 2000 reagent (Invitrogen, CA) in Opti-MEM media (Invitrogen, CA) for 18 hours after which they were adhered to the plate overnight before different immune effectors at 1∶1 Effector to target ratios were added. The cells were then lysed with lysis buffer and the relative Luciferase activity was measured using the Luciferase assay reagent kit obtained from Promega (Madison, WI).

### Statistical analysis

An unpaired, two-tailed student t- test was performed for the statistical analysis. One way ANOVA with a Bonferroni post test was used to compare the different groups.

## Results

### MSCs and DPSCs were selected based on their phenotypic and functional properties

MSCs were CD166^+^CD105^+^CD99^+^CD34^−^CD45^−^and CD14^−^ based on the flow cytometric analysis (data not shown). In addition, MSCs were capable of differentiating to osteogenic, chondrogenic and adipogenic lineages (data not shown, www.clonetics.com). DPSCs were isolated as described previously [26] and is shown by us and by others [10] to have osteogenic, chondrogenic and adipogenic properties.

### IL-2 activated NK cells are potent inducers of cell death in MSCs

**Figure 1:**
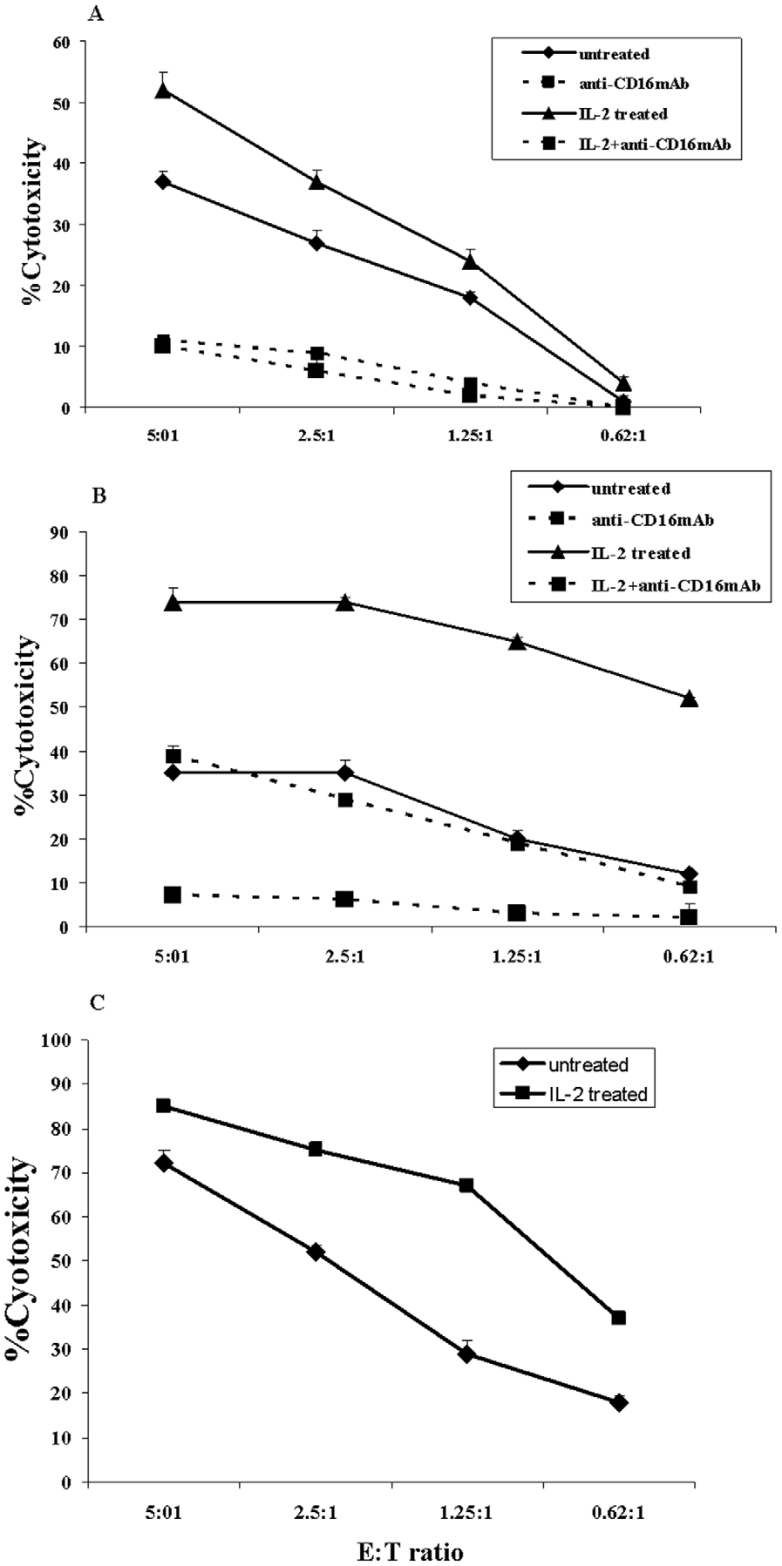
Potent lysis of MSCs and DPSCs by the Natural Killer cells; inhibition by anti-CD16 antibodies. Highly purified NK cells were left untreated or treated with anti-CD16 mAb (3 µg/ml), IL-2 (1000 u/ml), or a combination of IL-2 (1000 u/ml) and anti-CD16 mAb (3 µg/ml) for 8–12 hours before they were added to the ^51^Cr labeled MSCs (**Fig. 1A**) or ^51^Cr labeled DPSCs (**Fig. 1B**) or ^51^Cr labeled K562 (**Fig. 1C**) at different effector to target (E:T) ratios. After 4 hours of incubation the supernatants were removed and the released radioactivity was counted by a γ counter. % cytotoxicity was determined as indicated in the Materials and Methods section. One of five representative experiments is shown in this figure.

Highly purified human NK cells were treated with and without IL-2 for 8–12 hours before they were added to ^51^Cr labeled MSCs (Fig. 1A) or DPSCs (Fig. 1B) or NK sensitive K562s (Fig. 1C). The addition of freshly isolated untreated NK cells had lower cytotoxic activity against MSCs (Fig. 1A), DPSCs (Fig. 1B) and K562s (Fig. 1C). However, activation with IL-2 increased cytotoxicity substantially and resulted in a significant lysis of stem cells (p<0.05) (Fig. 1).

### Anti-CD16 antibody induces death in a subset of untreated and IL-2 treated NK cells and inhibits NK cell mediated lysis of MSCs and DPSCs

NK cells were left untreated or treated with anti-CD16 antibody and/or IL-2 for 8-12 hours before they were added to ^51^Cr labeled MSCs or DPSCs. As shown in a number of previous studies [18], [19], [20] and below, anti-CD16 mAb treatment induced death in a subset of NK cells and inhibited NK cell cytotoxicity against MSCs and DPSCs (p<0.05) (Fig. 1). The addition of the combination of IL-2 and anti-CD16 mAb treatment also induced death in a subset of NK cells and inhibited NK cell cytotoxicity against MSCs and DPSCs when compared to IL-2 activated NK cells (p<0.05) (Fig. 1).

### Monocytes prevent NK cell mediated lysis of MSCs and DPSCs

**Figure 2:**
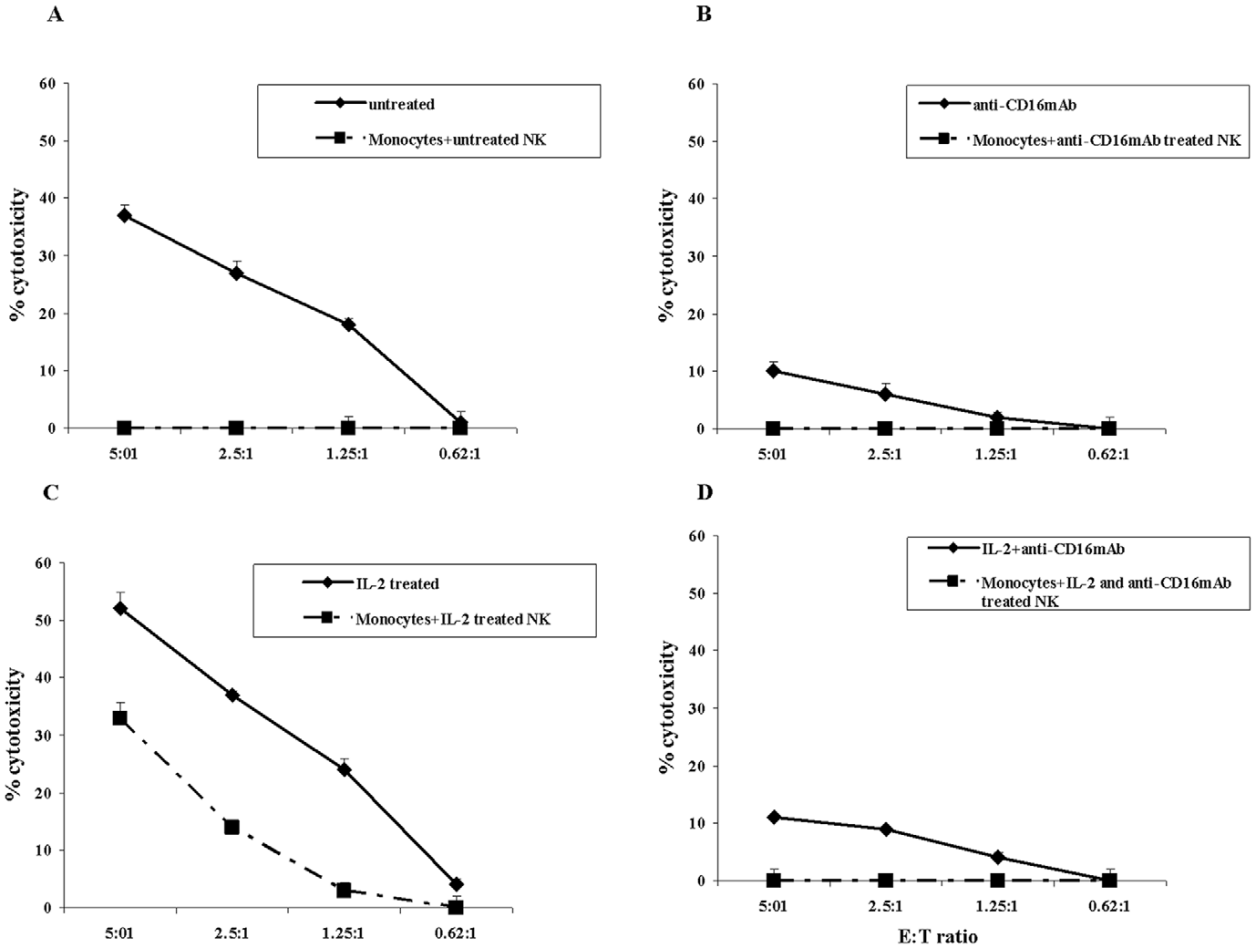
Decreased killing of MSCs by NK cells when MSCs were co-cultured with monocytes prior to the addition of NK cells. Monocytes were purified from PBMCs and irradiated as indicated in the Material and Methods section. MSCs (1×10^6^ cells/plate) were cultured with the irradiated monocytes (monocyte: MSC ratio of 1∶1) for 24–48 hours before they were removed from the plates, washed and labeled with ^51^Cr and used as targets in the cytotoxicity assays against NK cells. The NK samples were either left untreated (**Fig. 2A**) or treated with anti-CD16 mAb (3 µg/ml) (**Fig. 2B**), IL-2 (1000 u/ml) (**Fig. 2C**), or a combination of IL-2 (1000 u/ml) and anti-CD16 mAb (3 µg/ml) (**Fig. 2D**) for 24–48 hours before they were added to ^51^Cr labeled MSCs at different effector to target (E:T) ratios. Supernatants were removed after 4 hours of incubation and the released radioactivity counted by a γ counter. % cytotoxicity was determined as indicated in the Material and Methods section. One of three representative experiments is shown in this figure.

**Figure 3:**
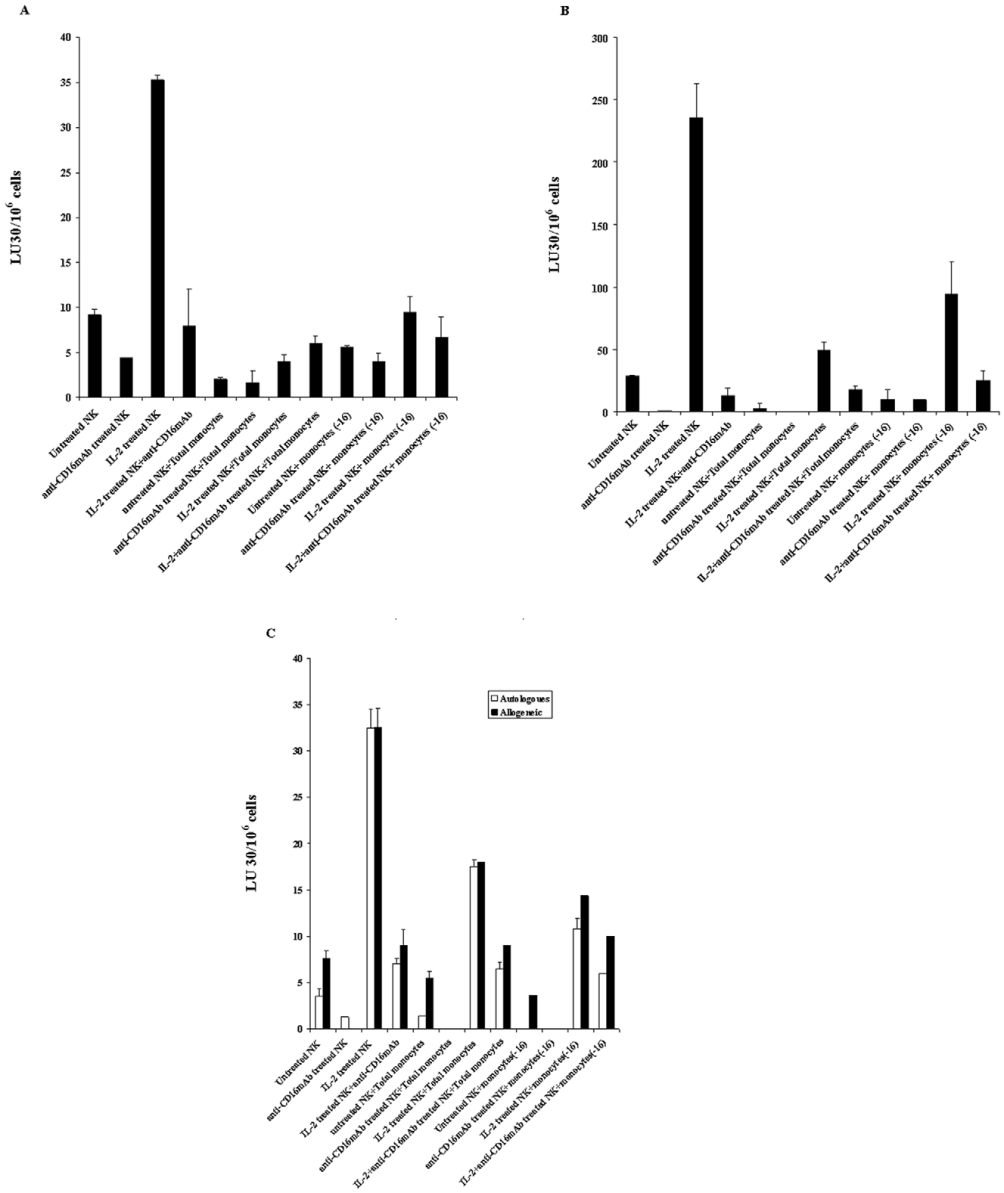
Total monocytes or those depleted of CD16^+^ subsets of monocytes protect MSCs and DPSCs against NK cell mediated cytotoxicity. MSCs (**Fig. 3A**) or DPSCs (**Fig. 3B**) each at 1×10^6^ cells/plate were co-cultured with highly purified and irradiated total monocytes or CD16^−^ subsets of monocytes at 1∶1 MSCs to monocyte ratio for 24–48 hours before they were detached, washed and labeled with ^51^Cr and added to untreated or IL-2 (1000 u/ml) pre-treated or anti-CD16mAb (3 µg/ml) pre-treated, or a combination of IL-2 (1000 u/ml) and anti-CD16 mAb (3 µg/ml) pre-treated NK cells at different E:T ratios. NK cells were pre-treated as indicated for 24–48 hours before they were added to the co-cultures of monocytes with MSCs. After 4 hours of incubation the supernatants were removed and the released radioactivity counted by a γ counter. % cytotoxicity was determined at different E:T ratio, and LU_30_/10^6^ cells were calculated using the inverse of the number of effectors needed to lyse 30% of the MSCs or DPSCs X100. Both autologous and allogeneic DPSCs (**Fig. 3C**) at 1×10^6^ cells/plate were cultured with highly purified and irradiated total monocytes or CD16- subsets of monocytes at 1∶1 DPSCs to monocytes for 24–48 hours before they were detached, washed and labeled with ^51^Cr and added to untreated or IL-2 (1000 u/ml) pre-treated or anti-CD16mAb (3 µg/ml) pre-treated, or a combination of IL-2 (1000 u/ml) and anti-CD16 mAb (3 µg/ml) pre-treated NK cells at different E:T ratios. NK cells were pre-treated as indicated for 24–48 hours before they were added to the co-cultures of monocytes with DPSCs. After 4 hours of incubation the supernatants were removed and the released radioactivity counted by a γ counter. % cytotoxicity was determined, and LU_30_/10^6^ cells were calculated using the inverse of the number of effectors needed to lyse 30% of DPSCs X100.

Monocytes were purified from PBMCs and irradiated as indicated in the Material and Methods section. MSCs and DPSCs were co-cultured with allogeneic irradiated monocytes for 24–48 hours before they were labeled with ^51^Cr and used in the cytotoxicity assays against NK cells. NK cells were left untreated or pre-treated with anti-CD16 antibody and/or IL-2 for 24–48 hours before they were used in the cytotoxicity assays against MSCs and DPSCs. The addition of monocytes to MSCs significantly protected the MSCs (Fig. 2 and 3A) and DPSCs (Fig. 3B) from NK cell mediated cytotoxicity (p<0.05). Significant inhibition of NK cell cytotoxicity by monocytes could be observed against untreated and IL-2 treated NK samples (p<0.05) (Figs. 2 and 3). Monocytes also increased the levels of alkaline phosphatase staining in MSCs and prevented decrease in alkaline phosphatase expression induced by IL-2 activated NK cells (data not shown). Untreated or anti-CD16 antibody treated live or irradiated monocytes were not able to mediate cytotoxicity against MSCs, DPSCs or K562 cells (data not shown). Overall, these experiments indicated that monocytes protect MSCs and DPSCs against NK cell mediated lysis. The reason cytotoxicity was shown both as percentage of killing over different E:T ratios (Figs. 1 and 2) and as Lu30/10^6^ cells (Fig. 3) is to demonstrate the clear relationship between cytotoxicity and calculated lytic units which will be used from here on after.

### Both the total populations of monocytes and those depleted of CD16^+^ subsets prevented NK cell mediated cytotoxicity against MSCs and DPSCs

Since unbound anti-CD16 antibody or shed antibody from the NK cells could affect the small subpopulation of CD16^+^ monocytes by modulating their function, we opted to sort out this small subset of monocytes from the total population of monocytes (16^−^) and compared their function to the activity of monocytes containing both the CD16^+^ and CD16^−^ subpopulations (total population) for the prevention of NK cell mediated cytotoxicity against MSCs and DPSCs. Both total population and CD16^+^ depleted subsets of monocytes could significantly prevent NK cell mediated lysis of MSCs (Fig. 3A) and DPSCs (Fig. 3B and 3C). Similar results were obtained in regards to the protection of DPSCs by monocytes against NK cell mediated lysis when the function of NK cells and monocytes was assessed with both allogeneic and autologous DPSCs (Fig. 3C). Furthermore, the removal of CD16 antibody by extensive washings of NK cells before their culture with monocytes and stem cells had similar results to those which were left unwashed and used in co-cultures with monocytes and stem cells (data not shown).

### Monocytes prevent anti-CD16 antibody mediated NK cell death

**Figure 4:**
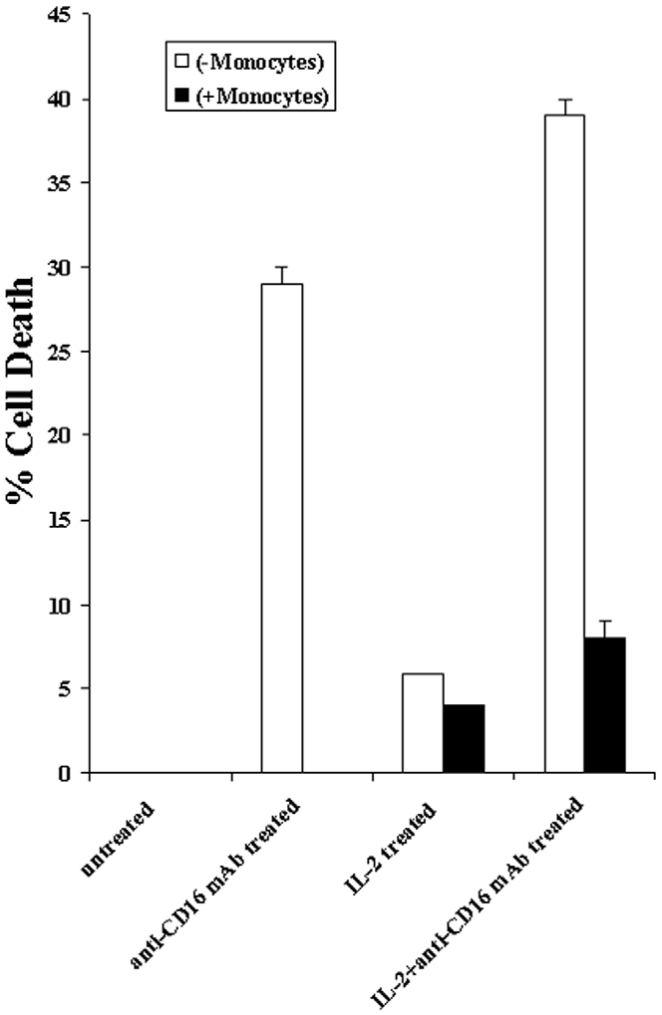
Monocytes prevent NK cell death induced by anti-CD16mAb. NK cells were labeled with FITC before they were either left untreated or treated with anti-CD16 mAb (3 µg/ml), IL-2 (1000 u/ml), or a combination of IL-2 (1000 u/ml) and anti-CD16 mAb (3 µg/ml) and immediately added to monocytes at 1∶1 (NK: monocyte) ratios. After an overnight incubation the levels of cell death in NK cells were determined by flow cytometric analysis of Propidium Iodide stained cells. FITC labeled NK cells were gated to determine the levels of PI stained NK cells. 10,000 events were analyzed for each sample. One of three representative experiments is shown in this figure.

Since monocytes were capable of protecting MSCs and DPSCs from NK cell mediated lysis we reasoned that they may also protect NK cells from undergoing cell death. Therefore, to investigate the protection of NK cells by monocytes, untreated or IL-2 treated and/or anti-CD16mAb treated NK cells were co-cultured with monocytes immediately after purification and treatment, and their viability was determined after an overnight incubation. As shown in Fig. 4 monocytes prevented NK cell death induced by anti-CD16 antibody in the presence and absence of IL-2 treatment (p<0.001). Therefore, monocytes are potent inducers of NK cell survival.

### Monocytes increase IFN-γ, IL-6, TNF-α and VEGF secretion substantially when cultured with and without NK cells and MSCs or NK cells and DPSCs

**Figure 5:**
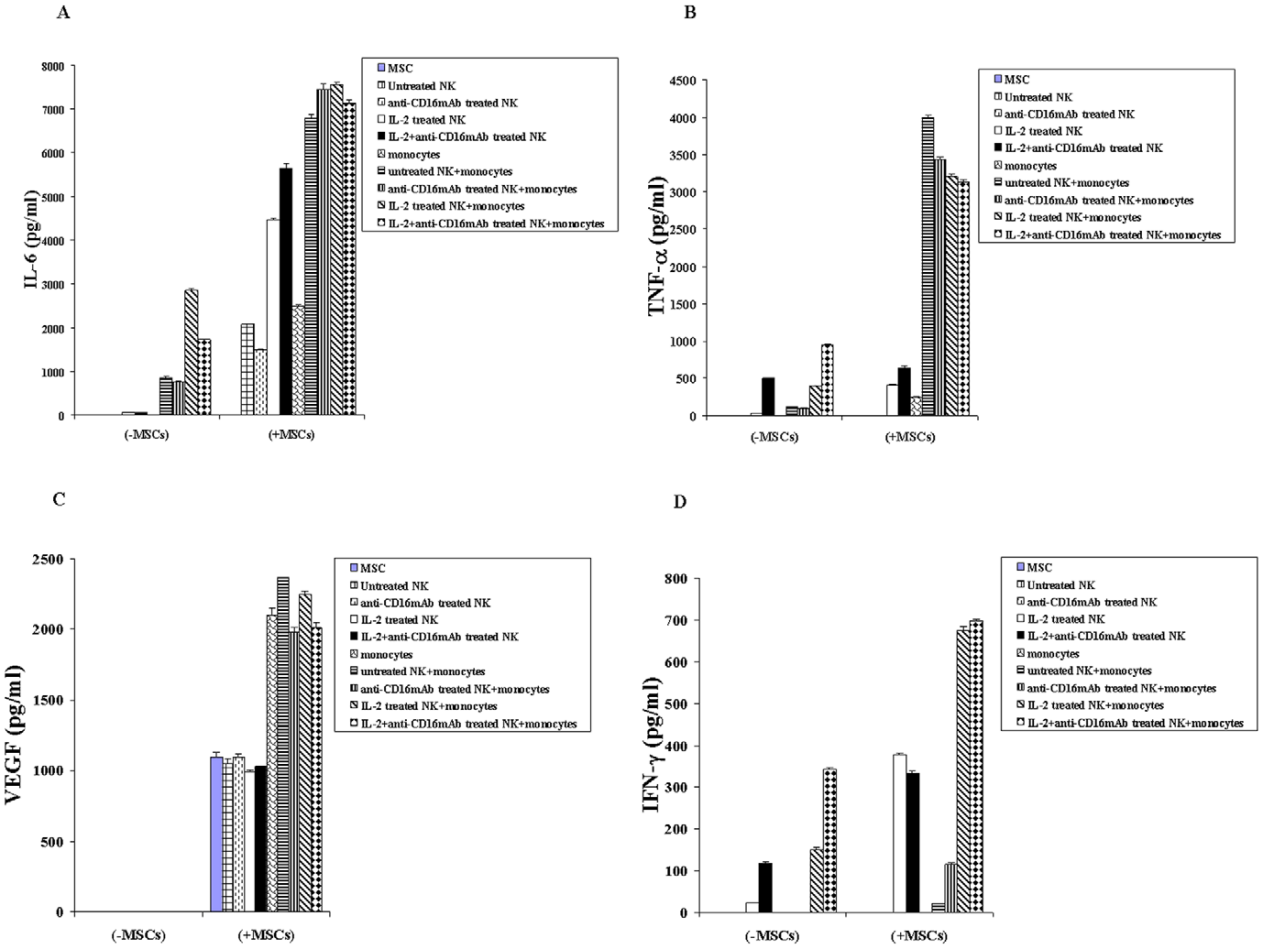
Monocytes synergize with NK cells and MSCs to induce IL-6, TNF-α, VEGF and IFN-γ. MSCs (1×10^5^ cells/well) were co-cultured with and without irradiated Monocytes at 1∶1 MSCs to monocytes for 24–48 hours before untreated or IL-2 (1000 u/ml) pre-treated or anti-CD16mAb (3 µg/ml) pre-treated, or a combination of IL-2 (1000 u/ml) and anti-CD16 mAb (3 µg/ml) pre-treated NK cells at 1∶1 NK: monocyte ratios were added to the MSC: moncyte co-cultures. NK cells were pre-treated as indicated for 24–48 hours before they were added to the co-cultures of monocytes and MSCs. NK samples were also cultured in the absence of monocytes and MSCs. After 24–48 hours of the addition of NK cells the supernatants were removed from the cultures and the levels of IL-6 (**Fig. 5A**), TNF-α (**Fig. 5B**), VEGF (**Fig. 5C**), and IFN-γ (**Fig. 5D**) were determined using multiplex cytokine array kit. The results were also confirmed for each individual cytokine using single ELISAs (data not shown). One of three representative experiments is shown in this figure.

Irradiated monocytes were added to MSCs for 24–48 hours before the addition of untreated or IL-2 pre-treated and/or anti-CD16 antibody pre-treated NK cells. After 24–48 hours of incubation with NK cells the supernatants were removed, and the levels of IFN-γ, IL-6, TNF-α and VEGF secretion were determined using both multiplex cytokine array assay and single ELISAs (Fig. 5). Monocytes increased the secretion of IL-6 and TNF-α in co-cultures with either NK cells or with MSCs (p<0.05) (Figs. 5A and 5B). However, the highest increase was obtained when monocytes were added to NK and MSCs (p<0.05) (Figs. 5A and 5B). As expected, IL-2 increased the secretion of TNF-α and IL-6 moderately in NK cells and the combination of IL-2 and anti-CD16 mAb had synergistic effect on the release of TNF-α by the NK cells (Figs. 5A and 5B). The addition of monocytes increased synergistically the secretion of IL-6 and TNF-α in all treated NK samples (p<0.05) (Figs. 5A and 5B). MSCs induced synergistically the secretion of IL-6 when cultured with NK samples (p<0.05) (Fig. 5A). Secretion of IL-6 and TNF-α were significantly augmented when monocytes were added to MSCs and NK cells when compared to those in the absence of monocytes (p<0.05) (Figs. 5A and 5B). However, the levels of IL-6 and TNF-α reach plateau when monocytes were added to untreated or anti-CD16 mAb and/or IL-2 treated NK cells and MSCs (Figs. 5A and 5B).

**Figure 6:**
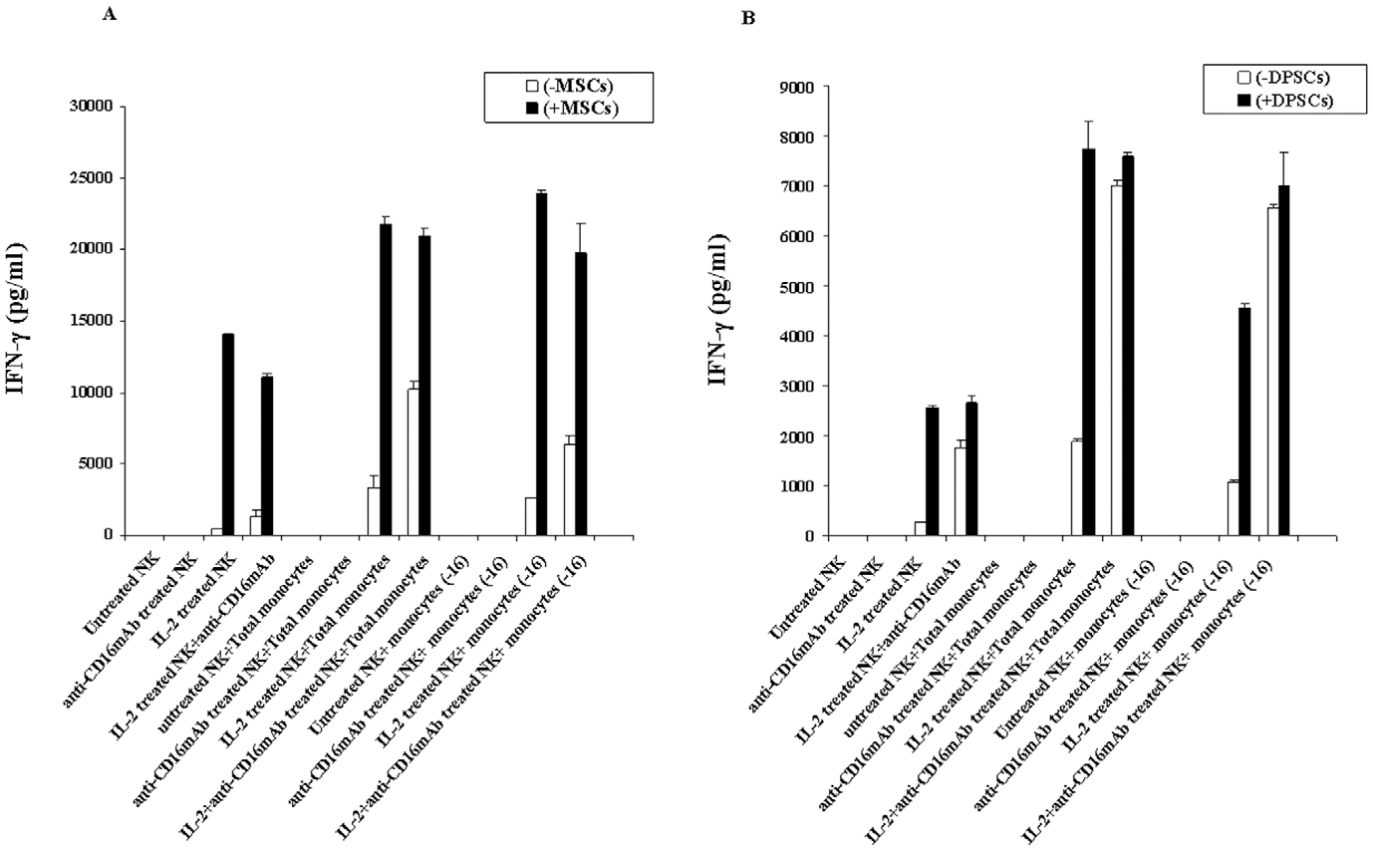
Total monocytes or CD16^−^ subsets of monocytes synergize with NK cells and MSCs or DPSCs to induce IFN-γ secretion. MSCs (**Fig. 6A**) or DPSCs (**Fig. 6B**) at 1×10^5^ cells/well were co-cultured with highly purified and irradiated total monocytes or CD16^−^ subsets of monocytes at 1∶1 MSCs or DPSCs to monocyte ratios for 24–48 hours before untreated or IL-2 (1000 u/ml) pre-treated or anti-CD16mAb (3 µg/ml) pre-treated, or a combination of IL-2 (1000 u/ml) and anti-CD16 mAb (3 µg/ml) pre-treated NK cells at 1∶1 MSCs or DPSCs to NK cells were added. NK cells were pre-treated as indicated for 24–48 hours before they were added to the co-cultures of monocytes and MSCs or DPSCs. After 24–48 hours of the addition of NK cells the supernatants were removed and subjected to specific ELISA for IFN-γ. One of four representative experiments is shown in this figure.

Secretion of VEGF was only observed in samples containing MSCs, and monocytes further increased secreted VEGF by MSCs (p<0.05) (Fig. 5C). Finally, as expected IL-2 treated NK cells secreted moderate amounts of IFN-γ which were synergistically increased when co-cultured in the presence of MSCs (p<0.05) (Fig. 5D). The addition of anti-CD16 mAb in combination with IL-2 to NK cells in the absence of MSCs increased secretion of IFN-γ when compared to IL-2 alone treated NK cells in the absence of MSCs. IFN-γ secreted levels remained similar between IL-2 alone and IL-2 and anti-CD16 mAb treated NK cells cultured with MSCs (Fig. 5D). Monocytes added to IL-2 or IL-2 and anti-CD16 antibody treated NK cells in the absence of MSCs or those in the presence of MSCs, synergistically increased the levels of secreted IFN-γ (p<0.05) (Fig. 5D). However, the highest increase in IFN-γ release was seen when monocytes were added to IL-2 or IL-2 and anti-CD16 mAb treated NK cells with MSCs (Fig. 5D). These results indicated that monocytes increased all of the above-mentioned cytokines in co-cultures with MSCs, and further synergized with IL-2 or IL-2 and anti-CD16 mAb treated NK samples to increase the release of cytokines in the co-cultures of NKs and MSCs. Similar results were obtained when NK cells were co-cultured with monocytes and DPSCs (Fig. 6).

### Total populations of monocytes or those depleted of CD16^+^ subset trigger significant release of IFN-γ by NK cells in a three way interaction with MSCs or DPSCs

Purified irradiated total monocytes or those depleted of CD16^+^ subsets were each co-cultured with MSCs or DPSCs for 24–48 hours before untreated or IL-2 and/or anti-CD16 mAb pre-treated NK cells were added. The incubation was then continued for another 24–48 hours before the supernatants were removed and subjected to specific ELISAs for IFN-γ (Fig. 6). The differences in secretion of IFN-γ between the two monocyte samples were either moderate or very slight when NK, monocytes and MSCs were all present in the co-cultures (Figs. 6A and 6B). Monocytes cultured with or without MSCs (Fig. 6A) and DPSCs (Fig. 6B) increased secretion of IFN-γ substantially when added to IL-2 or IL-2 and anti-CD16 antibody treated NK cells. Since monocytes alone or monocytes cultured with MSCs or DPSCs did not have any effect on IFN-γ secretion in all the experiments tested, we did not include them in Fig. 6 (data not shown).

### Protection of DPSCs from NK cell mediated cytotoxicity could still be seen when monocytes were sorted out from DPSCs before they were exposed to NK cells

**Figure 7:**
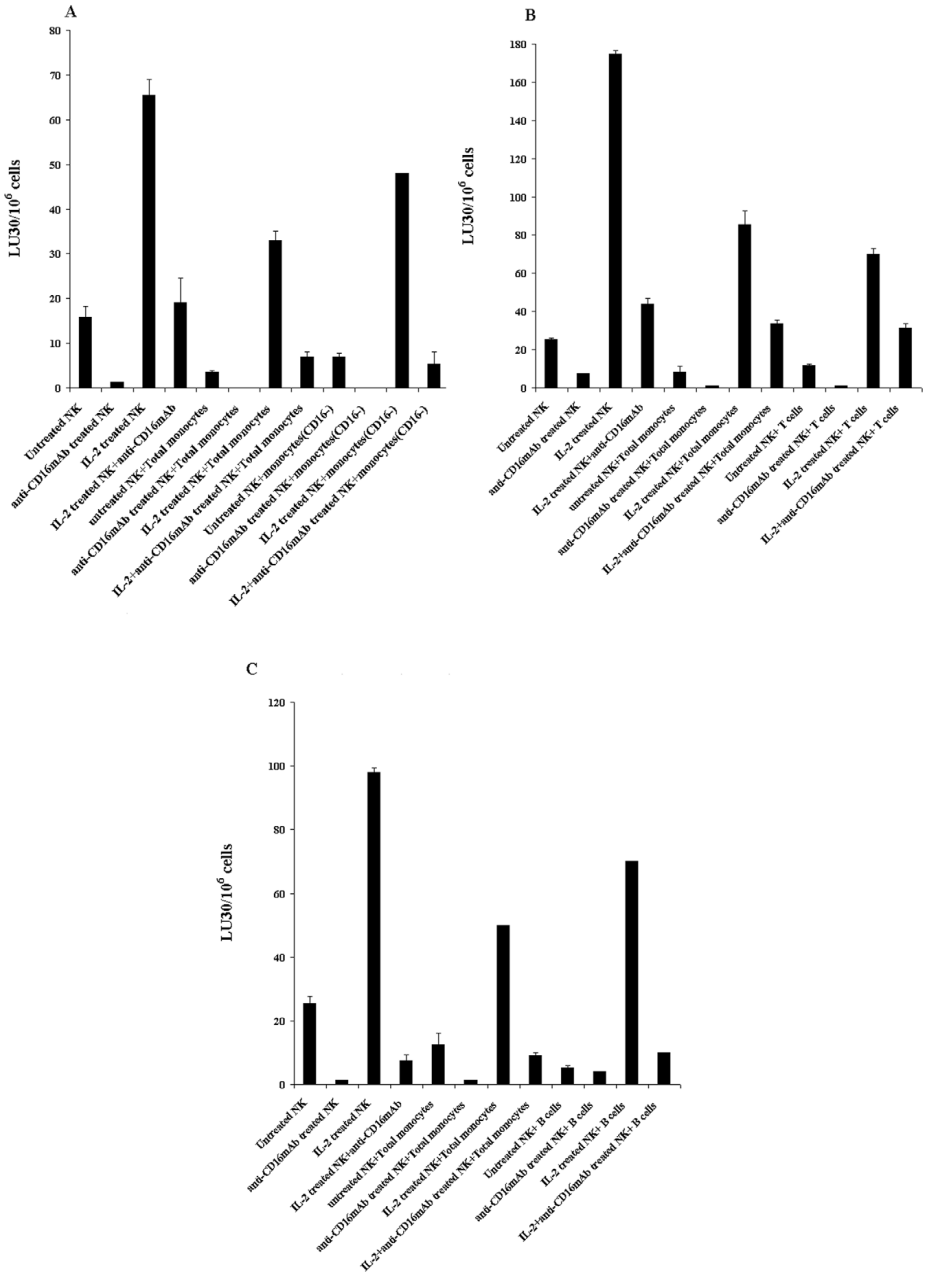
Decreased lysis of DPSCs by NK cells were seen when monocytes, T cells and B cells were sorted out from co-cultures of DPSCs with these cells before the addition of NK cells. Purified total population of monocytes or those depleted of CD16^+^ subsets (**Fig. 7A**), purified T cells (**Fig. 7B**) and purified B cells (**Fig. 7C**) were each co-cultured with 1×10^6^ DPSCs per plate at 1∶1 ratios for 24–48 hours, before the subsets of monocytes or T cells or B cells were sorted out and removed from DPSCs. Sorted DPSCs from monocytes, T cells and B cells were then labeled with 51Cr, washed and added to untreated or IL-2 (1000 u/ml) pre-treated or anti-CD16mAb (3 µg/ml) pre-treated, or a combination of IL-2 (1000 u/ml) and anti-CD16 mAb (3 µg/ml) pre-treated NK cells. After 4 hours of incubation the supernatants were removed and the released radioactivity counted by a γ counter. % cytotoxicity was determined at different E:T ratio, and LU_30_/10^6^ cells were calculated using the inverse of the number of effectors needed to lyse 30% of the DPSCs X100.

Purified total population of monocytes or those depleted of CD16^+^ subsets were each co-cultured with DPSCs for 24–48 hours, before they were sorted out and removed from DPSCs. DPSCs were then labeled with ^51^Cr and cultured with untreated or IL-2 and/or anti-CD16 mAb pre-treated NK cells. As shown in Fig. 7A, protection from NK cell mediated lysis could still be seen when monocytes were removed from DPSCs before their exposure to NK cells. However, this effect was not specific to monocytes alone since the addition of either T cells (Fig. 7B) or B cells (Fig. 7C) to DPSCs and their removal after 24–48 hours were also protective against NK cell mediated lysis of DPSCs.

### Monocytes and T cells are targets for NK cell mediated lysis

**Figure 8:**
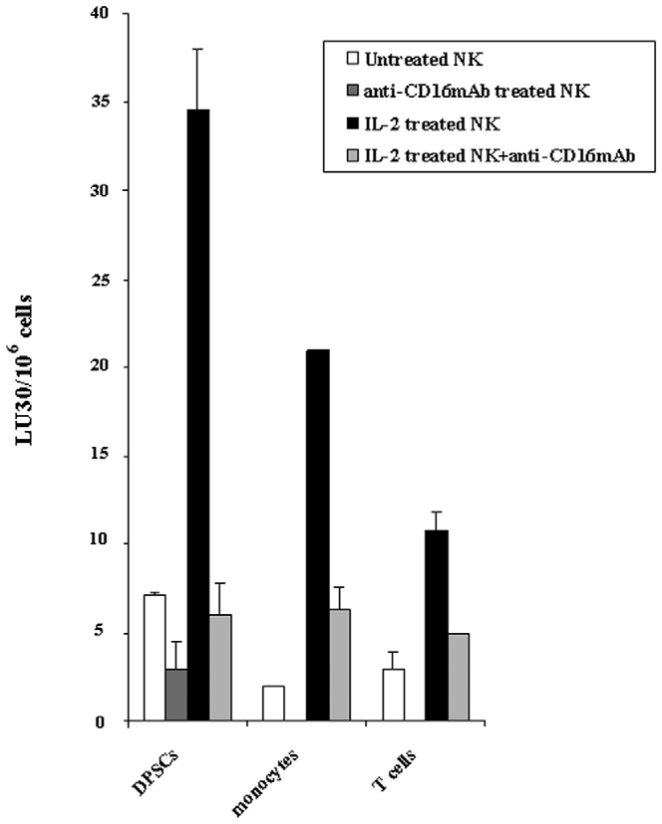
Monocytes and T cells are targets for NK cell mediated lysis. Highly purified NK cells were left untreated or treated with anti-CD16 mAb (3 µg/ml), IL-2 (1000 u/ml), or a combination of IL-2 (1000 u/ml) and anti-CD16 mAb (3 µg/ml) for 8–12 hours before they were added to the ^51^Cr labeled DPSCs, or ^51^Cr labeled monocyts or ^51^Cr labeled T cells at different effector to target (E:T) ratios. After 4 hours of incubation the supernatants were removed and the released radioactivity counted by a γ counter. % cytotoxicity was determined at different E:T ratio, and LU_30_/10^6^ cells was calculated using the inverse of the number of effectors needed to lyse 30% of either DPSCs, monocytes or T cells X100. One of three representative experiments is shown in this figure.

**Figure 9:**
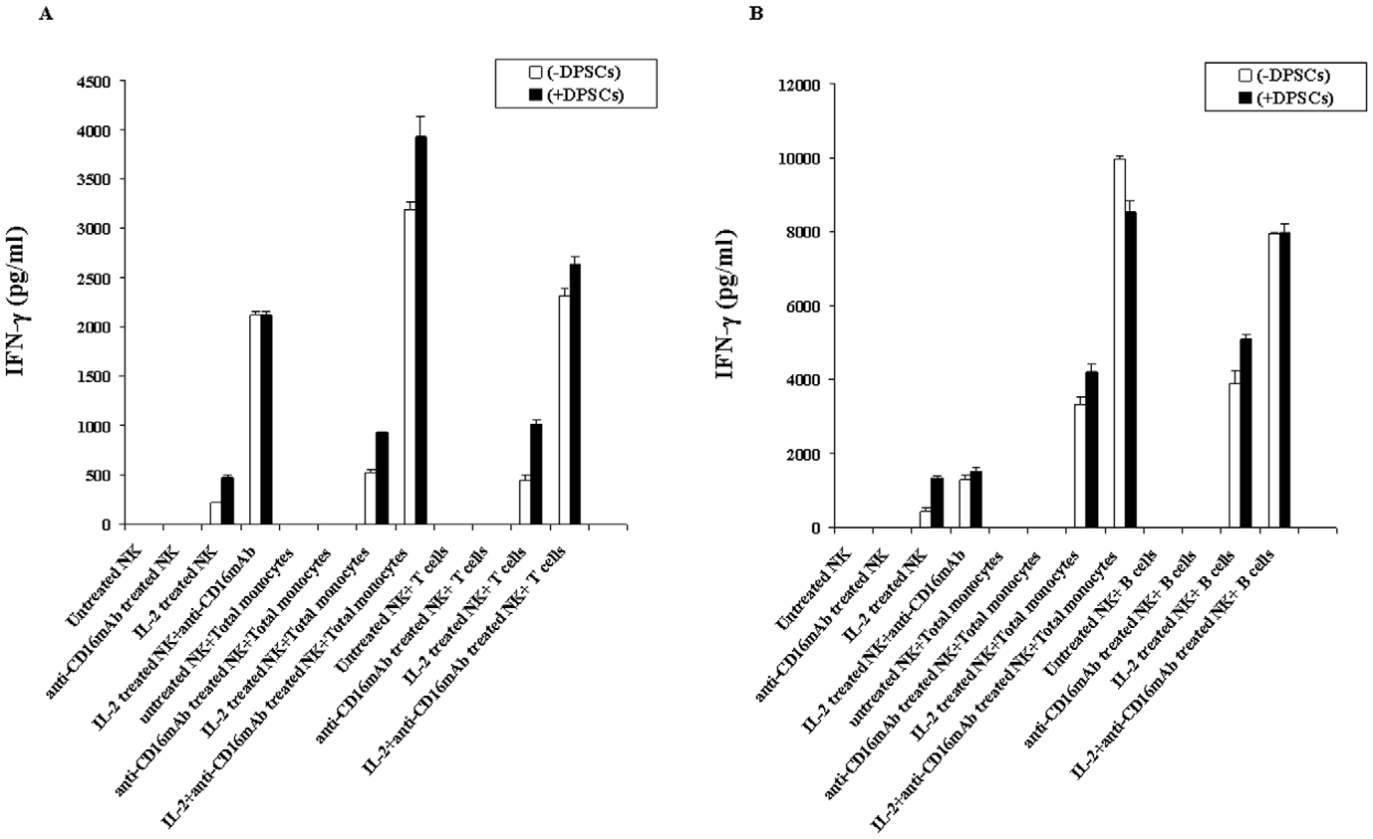
Total monocytes or T cells or B cells synergize with NK cells and DPSCs to induce IFN-γ secretion. DPSCs (1×10^5^ cells/well) were co-cultured with highly purified total monocytes (**Fig. 9A and 9B**) or T cells (**Fig. 9A**) or B cells (**Fig. 9B**) at 1∶1 DPSCs to monocyte or T cell or B cell ratios for 24–48 hours before untreated or IL-2 (1000 u/ml) pre-treated or anti-CD16mAb (3 µg/ml) pre-treated, or a combination of IL-2 (1000 u/ml) and anti-CD16 mAb (3 µg/ml) pre-treated NK cells at 1∶1 DPSCs to NK cells were added. NK cells were pre-treated as indicated for 24–48 hours before they were added to the co-cultures of monocytes or T cells or B cells and DPSCs. After 24–48 hours of the addition of NK cells the supernatants were removed and subjected to specific ELISAs for IFN-γ. One of four representative experiments is shown in this figure.

Since significant IFN-γ secretion could also be obtained in the co-cultures of IL-2 treated NK cells with monocytes we determined whether they were also targets of NK cell mediated lysis. As shown in Fig. 8 both monocytes and T cells were targets of NK cell mediated lysis. In accordance, significant IFN-γ secretion could also be obtained in supernatants removed from co-cultures of NK cells with monocytes (Fig. 9A) or T cells (Fig. 9A) or B cells (Fig. 9B). One distinction was in the magnitude of secreted IFN-γ by the NK cells treated with IL-2 and anti-CD16 mAb in which those cultured with monocytes secreted higher levels than those cultured with either T cells or B cells (Fig. 9).

### Monocytes are major inducers of NFκB activity in epithelial cells

**Figure 10:**
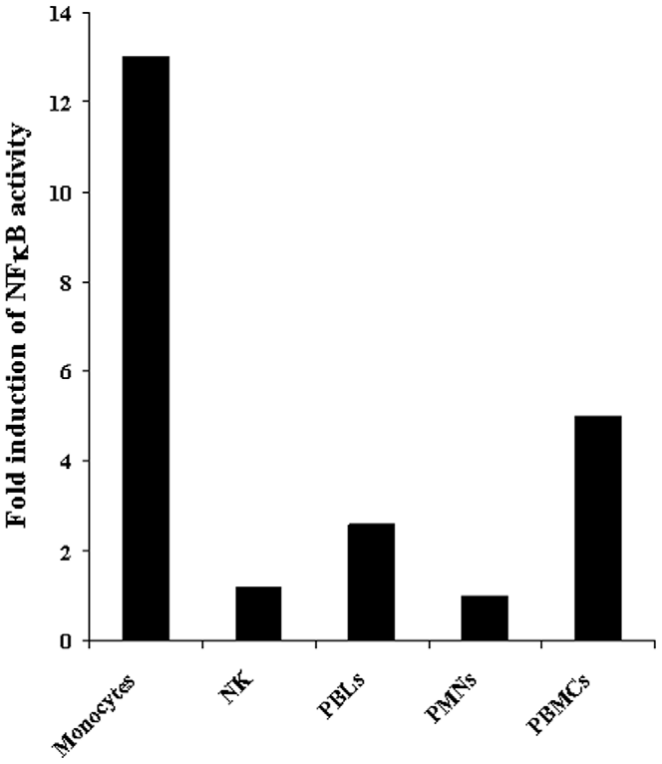
Monocytes are potent inducers of NFκB activity in HEp2 cells. HEp2 cells were transfected with NFκB luciferase reporter vector before they were co-cultured with monocytes, purified NK cells, peripheral blood lymphocytes (PBLs), polymorphonuclear (PMNs) and peripheral blood mononuclear cells (PBMCs) at 1∶1 ratio. After 4 hours of incubation the fold induction of NFκB activity in the samples was determined over the control HEp2 cells in the absence of immune effectors.

Purified subpopulations of the immune effectors, namely, highly purified monocytes, Natural Killer cells, PMNs and total populations of peripheral blood mononuclear cells (PBMCs) and peripheral blood lymphocytes (PBLs) were each co-cultured with NFκB reporter transfected HEp2 cells (Fig. 10), and the levels of NFκB activity were determined after 4 hours of incubation. As shown in Fig. 10 monocytes induced the highest increase in NFκB activity whereas all the other populations including NK cells had minimal or no effect on NFκB activity (p<0.05).

## Discussion

Successful transplantation of stem cells in an immunocompetent host will eventually depend on the ability of transplanted stem cells to survive during their interaction with the host immune effectors. Although a number of previous studies have attributed a lack of immunogenicity to stem cells, others have shown significant lysis of these cells by the cytotoxic immune effectors [4], [6], [7]. Therefore, detailed analysis of immune cell interaction with stem cells is important, and should provide the basis for designing novel strategies to increase the viability and function of stem cells in different tissue engineering applications. It is therefore, important to determine the potential mechanisms by which stem cells can be protected from the lysis by cytotoxic immune effectors.

To study the detailed function of cytotoxic immune effectors against stem cells we purified human NK cells and studied their effect on MSCs and DPSCs. In addition, we determined the interaction of NK cells with other subsets of immune effectors during their interaction with stem cells. In agreement with previous studies we also show in this report that NK cells can target and kill MSCs significantly. Indeed, when compared to K562 lysis, NK cells can also mediate significant lysis of MSCs (Figs. 1 and 2) and DPSCs. Moreover, we demonstrate for the first time that DPSCs are also targets of NK cell mediated killing, and that stem cells in general have exquisite sensitivity to NK cell mediated lysis. In support of this notion, we have recently determined that human embryonic stem cells are the most sensitive stem cells to NK cell mediated cytotoxicity when compared to DPSCs (manuscript in preparation). In addition, autologous DPSCs are as good of a target for NK cell mediated lysis as the allogeneic DPSCs.

As expected, IL-2 treated NK cells lysed a much higher percentage of MSCs and DPSCs when compared to untreated NK cells, although a range of 20% to 70% cytotoxicity could be observed by untreated NK cells depending on the donor NK cells used to lyse stem cells (data not shown). In addition, anti CD16 antibody treated NK cells lost their cytotoxic function against MSCs and DPSCs since this treatment resulted in anergy as well as the death of a subset of NK cells and prevention of cytotoxicity against MSCs and DPSCs. Moreover, significant induction of IFN-γ secretion could be observed in supernatants removed from the co-cultures of either IL-2 or IL-2 and anti-CD16 mAb treated NK cells with MSCs and DPSCs.

When MSCs and DPSCs were cultured with either viable or irradiated monocytes before they were exposed to IL-2 treated NK cells a significant decrease in NK cell mediated cytotoxicity could be observed. Interestingly, significant lysis of MSCs and DPSCs by untreated NK cells was also significantly and reproducibly blocked by the addition of monocytes (Fig. 2). To determine whether decreased lysis of stem cells by NK cells was due to competitive lysis of monocytes by NK cells we performed several experiments. Indeed, we confirmed that monocytes were also lysed by NK cells significantly. In addition, we found not only monocytes but also T and B cells to be significant targets of NK cell mediated killing. Furthermore, when we co-cultured stem cells with monocyte subsets and sorted to remove the monocytes from the stem cells we could still observe significant inhibition of NK cell mediated lysis, arguing against the protection of stem cell lysis by NK cells being solely on the bases of competitive lysis of monocytes. Therefore, even though lysis of monocytes by the NK cells may in part contribute to the prevention of NK cell lysis of stem cells, interaction of monocytes with stem cells can also provide resistance of stem cells against NK cell cytotoxicity. However, this function was not specific to monocytes since the addition of T and B cells to stem cells and their removal by sorting before their culture with NK cells could also provide resistance of stem cells against NK cell mediated cytotoxicity. Therefore, it is likely that interaction of NK cells with stem cells in the presence of other immune effectors is complex and it is governed by not only the ability of NK cells to lyse other immune effectors, but also due to increased resistance of stem cells to NK cell lysis after interaction with subsets of immune effectors.

Decrease in NK cell lysis of MSCs and DPSCs was paralleled with a significant induction of IFN-γ in a stepwise manner. Indeed, when MSCs or DPSCs were cultured with IL-2 treated NK cells alone we could observe significant induction of IFN-γ secretion. However, the highest increase was seen when NK cells were cultured with MSCs or DPSCs in the presence of monocytes. Therefore, although decreased killing of stem cells by the NK cells could be observed in the presence of monocytes, synergistic secretion of IFN-γ by the NK cells in the presence of monocytes and stem cells could be observed, indicating a discordant relationship between cytotoxicity and IFN-γ secretion. This was similar to the profiles which we had seen when NK cells were treated with IL-2 and anti-CD16 antibody in which significant decrease in cytotoxicity of NK cells could be observed in parallel with increased secretion of IFN-γ (Fig. 5, 6 and 9). We have previously shown that IL-2 rescues NK cells from anti-CD16 mAb mediated apoptosis [19], therefore, it is possible that monocytes also contribute to rescue from apoptosis and increase in secretion of IFN-γ by the NK cells. Indeed, monocytes prevent anti-CD16 mediated NK cell apoptosis (Fig. 4). Rescue of NK cells from apoptosis could be an important function of monocytes since 1- monocytes are known to increase NFκB activity in the cells, and 2-lysis of monocytes by the NK cells could result in activation and increased resistance of NK cells to cell death. In this respect T and B cells can also provide increased survival of NK cells since they too are targets of NK cell killing, but they are not capable of inducing NFκB in epithelial cells to the levels which were induced by monocytes. In addition, even though T and B cells could cause resistance of stem cells to NK cell mediated cytotoxicity, they could not raise the levels of IFN-γ secretion by the IL-2 and anti-CD16 mAb treated NK cells to the levels which were observed when these cells were cultured with monocytes and stem cells (Fig. 9).

These observations prompted us to speculate regarding the significance of interaction of monocytes with NK cells and stem cells. It is plausible that monocytes serve as not only effectors which provide survival for NK cells and stem cells but also they may serve as shields against NK cell lysis of stem cells. Similar to anti-CD16 antibody mediated effect on IL-2 treated NK cells, monocytes too can shield stem cells from killing by the NK cells by increasing the total IFN-γ release by the NK cells while decreasing the cytotoxic function of NK cells (split anergy), resulting in an increased protection and differentiation of stem cells. Indeed, monocytes also increased TNF-α, IL-6 and VEGF secretion in the co-cultures of stem cells with NK cells which could further augment induction of NFκB and increased differentiation of stem cells. The shielding effect of monocytes could be a more generalized function of immune effectors since NK cells can also target T and B cells. This may have significant implications regarding the role of NK cells in not only limiting inflammation, but also the significance of other immune effectors in shielding and limiting the cytotoxic function of NK cells against stem cells in order to raise maximally the secretion of key cytokines for speedy and optimal differentiation of stem cells during inflammation. This is precisely what is observed in cancer patients in whom global decrease in NK, cytotoxic T cells and monocytes have all been reported [29]. In addition, the increase in IFN-γ secretion by NK cells should provide the initial steps in increasing antigen specific functions of T and B cells and initiation of adaptive immunity.

We have recently observed an NFκB independent rescue of oral epithelial cells by monocytes (manuscript in prep). Therefore, it is likely that monocytes elevate survival of MSCs and DPSCs through NFκB dependent and independent pathways. In addition, both the total population of monocytes and those depleted of CD16^+^ subsets were able to increase NFκB and survival of oral epithelial cells (manuscript in prep) and MSCs resulting in an increase in cytokine secretion.

We have previously reported that rescue from cell death is governed by inverse induction of NFκB and JNK and decreased secretion of IgFbp6 in epithelial cells [26], [30]. [31]. Whether monocytes prevent stem cell death through similar mechanisms should await future investigation.

Our previous work demonstrated the decoupling of NK cell cytotoxicity from cytokine secretion [16]. The cytotoxic function of non-dissociated NK-tumor cell conjugates remained very low and did not increase when supplemented with IL-2 although they proliferated and secreted higher amounts of TNF-α and IFN-γ in the supernatants [16]. In contrast, NK cells dissociated from the target conjugates exhibited more cytotoxic activity, but they proliferated poorly and secreted lower levels of TNF-α or IFN-γ following IL-2 treatment. More importantly, a fraction of NK cells in non-dissociated NK-tumor conjugates were programmed for cell death by apoptosis [16]. Furthermore, the addition of intact and F(ab)'^2^ fragment of anti-CD16 antibodies in combination with IL-2 significantly and reproducibly inhibited the cytotoxic function of NK cells, but the NK cells secreted relatively higher amounts of IFN-γ [18], [19], [20]. As mentioned above, we observed a similar trend when monocytes were added to MSCs/DPSCs and the IL-2 treated NK cells. This function of anti-CD16 receptor antibody and monocytes therefore can be exploited to decrease rejection of stem cells by the NK cells. Indeed, for optimal engraftment of stem cells one may require to target both cytotoxic NK and T cells and in this regard the use of the combination of anti-CD16 antibody and OKT3 antibody should theoretically achieve this objective. We are currently using this strategy to improve engraftment of stem cells in pulp tissues.

Given the high sensitivity of stem cells to NK cell cytotoxicity, it is possible that significant elevation in NK cell signaling by MSCs and subsequent initiation of signaling for anergy and cell death of a subset of NK cells contribute to the observed suppression of NK cell cytotoxicity. Indeed, significant down modulation of NK cell receptors NKp30, NKp44 and NKG2D was also observed after interaction of NK cells with MSCs [6]. Receptor down-modulation could likely be a mechanism to limit the levels of signaling for the survival of the NK cells. Therefore, the suppression of NK cell cytotoxic function observed by MSCs could well be due to over-stimulation of NK cells by the stem cells and induction of cell death in NK cells. Therefore, the reason MSCs were considered to prevent auto-immunity may be due to their competitive binding and inactivation and removal of killer NK cells. These possibilities are currently under investigation in our laboratory.

Overall, studies reported in this paper provide evidence for an important function of monocytes in protection of MSCs and DPSCs from NK cell mediated lysis resulting in an increased secretion of key cytokines for optimal survival, proliferation and differentiation of stem cells in a three way interaction.
